# Improving peritoneal dialysis fluid culture-positivity yield from 2022 to 2023

**DOI:** 10.4102/sajid.v39i1.684

**Published:** 2024-11-29

**Authors:** Jenna A. van der Vyver, Teena Thomas

**Affiliations:** 1 Department of Clinical Microbiology and Infectious Diseases, Faculty of Health Science, School of Pathology, University of the Witwatersrand, Johannesburg, South Africa; 2 Infection Control Services Laboratory, National Health Laboratory Services, Johannesburg, South Africa

**Keywords:** dialysis bag, culture yield, peritonitis, peritoneal fluids, dialysis fluid, culture-positivity

## Abstract

**Background:**

Microbiological testing of peritoneal dialysis bags for peritonitis often yields culture-negative results. Culture-negative samples should not exceed > 15% according to the International Society for Peritoneal Dialysis. To reduce this issue, the addition of a blood culture bottle incubation step to the culture process was introduced at the Infection Control Services Laboratory (ICSL) of the National Health Laboratory Services (NHLS).

**Objectives:**

The aim of the study was to ascertain if the change in methodology increased the culture-positivity yield and reduced the culture-negative percentage.

**Method:**

Data from the NHLS Central Data Warehouse (CDW) were analysed to compare the culture-positive results over two periods: June–December 2022 when the non-blood culture (B/C) bottle method was used and January–July 2023 when the B/C bottle method was implemented.

**Results:**

The non-B/C culture method yielded a 23% culture-positivity yield, whereas the B/C bottle-based method yielded a 51% culture-positivity yield. However, the culture-negative yield for the B/C bottle-based method was high at 49%.

**Conclusion:**

The change in dialysis bag processing in 2023 led to a more than doubling in culture-positivity yield. However, the culture-negative percentage remained high. As a result, further modifications to the methodology are needed.

**Contribution:**

The study findings illustrate that the addition of the B/C bottle incubation step significantly improved peritoneal dialysis bag culture yields which directly impacts patient management.

## Introduction

The kidneys play an integral role in maintaining homeostasis in the human body, encompassing essential functions such as blood filtration, waste elimination, urine production, hormone regulation, red blood cell synthesis and acid-base balance.^1^ In cases of end-stage renal disease or renal failure, dialysis becomes imperative to sustain adequate kidney function.

Peritoneal dialysis (PD) uses the peritoneum as a natural filter for removal of bodily waste.^1^ While highly effective, PD may lead to complications such as hernia development, weight gain because of dextrose content in the dialysis fluid, treatment inefficacy and infections, notably peritonitis.^2^

The prevalence of PD-related peritonitis is up to 30% – 40% in patients during their course on dialysis.^3^ This type of peritonitis is a direct cause of death in 2% – 6% of patients and is a contributing factor in up to 10% – 20% of dialysed patients.^4^ In addition, peritonitis is a leading cause of hospitalisation, catheter loss and reason for change to haemodialysis.^5^ Early identification of the causative microorganism is crucial. Common causative agents include coagulase-negative staphylococci, coagulase-positive *Staphylococcus aureus*,^6^ other Gram-positive bacteria (such as Streptococcal spp.), occasionally Gram-negative bacteria and yeasts.^7^ Targeted antibiotic therapy is administered based on the organism cultured. Culture-negative peritonitis presents a huge challenge in the appropriate management of the affected patients.^8^

Culture-negative peritonitis is defined by the presence of cloudy dialysate and abdominal pain, with no microbiological growth even after 72 h.^8^ Contributing factors to culture-negative peritonitis encompass recent antibiotic exposure, low organism inoculum, inadequate culturing methods, or the presence of non-culturable or slow growing microorganisms like *Legionella, Campylobacter, Ureaplasma, Mycoplasma,* Enteroviruses or mycobacteria.^9^

Previously, the method for peritoneal fluid from dialysis bag processing for patients with suspected peritonitis at the Infection Control Services Laboratory (ICSL) involved direct sediment inoculation onto agar plates for culture.^10^ Comparative studies showed a suboptimal culture-positivity yield for this method. In one study comparing five processing methods including water lysis, direct inoculation, incubation into a blood culture bottle, Tween-80 incorporated blood agar and Triton-X treatment of the specimen for the diagnosis of peritonitis from PD bags, the ‘direct inoculation’ method had a culture-positivity yield of 46% while that of sample inoculation and incubation in a blood culture (B/C) bottle improved the yield to 58%.^11^

In another more recent study, incubating the sample in an aerobic B/C bottle until it flags positive, followed by plating out from the bottle onto agar plates saw a decrease in the culture-negative samples to 22.86%.^12^ Studies are aiming to reduce culture-negative yield based on the current International Society for Peritoneal Dialysis (ISPD) guidelines which recommend that culture-negative yield should be < 15% of all peritonitis episodes.^8^

This new culturing method of introducing the BacT/Alert Standard aerobic bottle into the sample processing steps was implemented at ICSL in January 2023.

Therefore, this study aimed to assess the difference in culture-positive yield from the old method (non-B/C bottle-based) of PD fluid processing to the new method (B/C bottle-based) introduced at the ICSL in January 2023.

## Research methods and design

### Peritoneal dialysis fluid culture process (non-blood culture bottle-based method)

The PD fluid processing procedure till December 2022 involved the following: the contents of the bag were mixed by inverting the bag 10 times to allow for homogenisation of the sample. Thereafter, the appearance of the dialysate fluid was recorded as clear, turbid, xanthochromic or bloody. Then, 2 mL of sample was aspirated from the bag for cell counts and antimicrobial substances to be tested. Additionally, two 50 mL aspirates from the bag were centrifuged at 3 000 rpm for 15 min. The sediment from the first one was used for microscopy (Gram stain and Ziehl-Neelsen stain), and the sediment from the second one was directly plated onto agar plates (blood, chocolate and MacConkey). The plates were incubated at 35°C ± 2°C for 48 h and assessed for culture growth daily over the 2-day period.

If culture growth occurred, further tests for organism identification and susceptibility testing followed.^13^

### Peritoneal dialysis fluid processing method (blood culture bottle-based method)

In January 2023, a modification to the processing method was introduced at ICSL. This method incorporated an additional step and use of the BacT/Alert aerobic B/C bottle as part of sample processing. This involved inoculating 5 mL of the sediment from one of the 50 mL centrifuged tubes into the B/C bottle. The B/C bottle was then incubated in the BacT/Alert instrument. Once the bottle flagged positive, sample was inoculated on agar plates (blood, chocolate and MacConkey) and incubated at 35°C ± 2°C for 48 h. Culture growth was evaluated daily for the 2 days. If culture growth occurred, further tests were performed as per the standard operating procedure document.^13^

### Data analysis

Data were received from the National Health Laboratory Services (NHLS). The requested dataset contained PD fluid culture results over two periods from June 2022–December 2022 and January 2023–July 2023. This dataset included ALL culture results. The culture-negative samples were denoted by the label ‘NG’ (no growth) under the culture result, signifying the absence of microbial growth. Conversely, culture-positive samples were denoted by organism identification.

As per the study objectives, the following formulas (Equation 1, Equation 2, and Equation 3) were used to determine results.

#### Percentage of culture-positivity yield for non-blood culture bottle-based method


$$\%\text{ of Culture-positive for non-B/C bottle-based method}=\frac{\text{Culture-positive number obtained from non-B/C bottle method}}{\text{Total number of dialysis bags processed by non-B/C bottle-based method}}\times 100$$ [Eqn 1]

#### Percentage of culture-positivity yield for blood culture bottle-based method


$$\%\text{ of Culture-positive for B/C bottle-based method}=\frac{\text{Culture-positive number obtained from B/C bottle}}{\text{Total number of dialysis bags processed by B/C bottle-based method}}\times 100$$ [Eqn 2]

#### Percentage difference of culture-positive yield of blood culture bottle method *versus* non-blood culture bottle method

Percentage difference of culture-positive yield (B/C bottle method vs. non-B/C bottle method) = (% of Culture-positive for B/C bottle-based method − % of Culture-positive for non-B/C bottle-based method)

#### Percentage of culture-negative yield for blood culture bottle method


$$\%\text{ of Culture-negative for B/C bottle-based method}=\frac{\text{Culture-negative number obtained from B/C bottle method}}{\text{Total number of dialysis bags processed by B/C bottle-based method}}\times 100$$ [Eqn 3]

### Ethical considerations

Ethical approval to conduct this study was obtained from the University of the Witwatersrand Human Research Ethics Committee (No. M230467).

Samples utilised in this study were sent for routine microbiological testing as part of patient management and therefore did not require participant consent. All patient identifiers such as name, age, race, gender, ID numbers, hospital numbers, among others were removed in order to maintain confidentiality of the data.

All procedures performed in studies involving human participants were in accordance with the ethical standards of the institutional and/or national research committee and with the 1964 Helsinki Declaration and its later amendments or comparable ethical standards.

## Results

**FIGURE 1:**
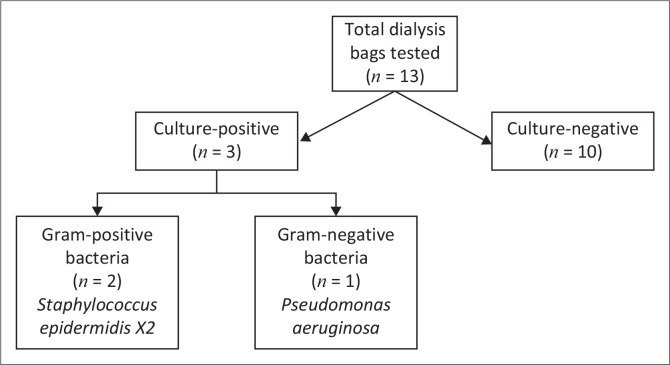
Non-blood culture bottle-based method results.

A total of 13 dialysis bags were tested using the non-B/C bottle-based method in 2022, of which 10 samples were culture-negative and 3 samples were culture-positive. Of the three culture-positive samples, two samples were Gram-positive bacteria (*Staphylococcus epidermidis*) and one sample cultured a Gram-negative bacterium (*Pseudomonas aeruginosa*) (Figure 1).

Using the formula previously stated in the data analysis section, the culture-positivity yield for non-B/C bottle-based method was 23.08%.

**FIGURE 2:**
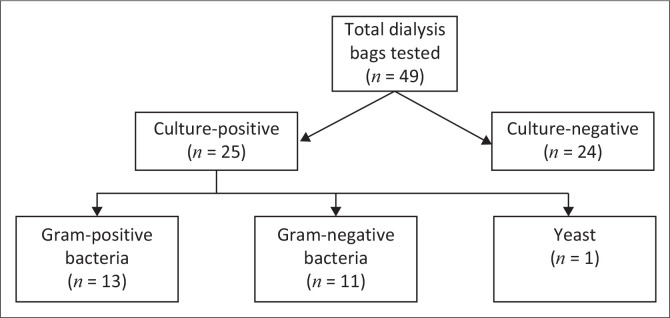
Blood culture bottle-based method results.

A total of 49 dialysis bags were tested using the B/C bottle-based method implemented in 2023, of which 24 samples were culture-negative and 25 samples were culture-positive. Of the 25 culture-positive samples, 13 samples were Gram-positive bacteria, 11 samples were Gram-negative bacteria and 1 sample was a yeast (Figure 2). Refer to Table 1 for the organism identification results of the B/C bottle-based method.

**TABLE 1:** Organisms cultured by the blood culture bottle-based method.

Organism cultured	No. of organisms cultured
**Gram-positive bacteria**	
*Staphylococcus epidermidis*	6
*Staphylococcus hominis*	1
*Staphylococcus haemolyticus*	1
*Streptococcus anginosis*	1
*Micrococcus* spp.	1
*Streptococcus mitis/oralis*	3
**Gram-negative bacteria**	
*Enterobacter cloacae*	1
*Pseudomonas fluorescens/putida*	2
*Acinetobacter* spp.	1
*Acinetobacter baumannii*	1
*Klebsiella pneumoniae*	5
*Escherichia coli*	1
**Yeast**	
*Candida glabrata*	1

Culture-positivity yield for the non-B/C bottle-based method was 23% and for the B/C bottle-based method was 51%. The difference in culture-positive yield between the two periods was 28%.

The culture-negative yield for the B/C bottle-based method was 49%.

## Discussion

This study showed that the newly introduced B/C bottle-based method for PD bag processing at ICSL improved the culture-positivity yield. The culture-positivity yield more than doubled from 2022 to 2023. However, the culture-negative yield for 2023, although improved from 2022 (77% culture-negative), remained higher than what is recommended. These findings agreed with a study where the culture-positivity yield increased; however, the culture-negativity did not meet the ISPD guidelines of < 15%.^12^

Some of the reasons for this high culture-negativity in our setting can be infection caused by *Mycobacterium tuberculosis*, which is of high prevalence in South Africa or infection because of other fastidious pathogens (as mentioned earlier).

### Limitations

There are a few limitations to this study. First of all, the same samples were not tested in the two time periods. A more precise evaluation of the culture-positivity yield of the two methodologies could have been achieved by concurrently testing the same dialysis bags. Another limitation of the study is that sample numbers in the first 7-month period were small. A larger sample size (> 30) would have ensured the results could have been compared for statistical significance. In addition, it was also unknown if empiric antibiotics were given to patients before sample collection which could increase culture-negative results.

### Recommendations

Although the study illustrated that the newly introduced B/C bottle-based method increased the culture-positivity rate in comparison to the direct inoculation method used previously, this study does leave room for future improvements and evaluations to be conducted. The study found that the culture-negativity yield was not compliant to the ISPD guidelines. Therefore, further interventions to improve on this are required. A study conducted in Thailand, in which 5 mL – 10 mL of the sediment was added to the B/C bottle had a lower culture-negativity yield (22.86%) than that of the ICSL.^12^ As a result, adding more than 5 mL of the inoculated sediment to the aerobic B/C bottle may further improve the culture-positivity yield. This notion was further supported by another study where 10 mL of the inoculated sediment was added to B/C bottles and culture-positivity increased to 73% while the culture-negativity rate reduced to 17%.^11^ By adding more quantity of the sediment into the B/C bottle, this could potentially lead to a further decrease in the culture-negative results. Additionally, where it is unknown if a patient received antibiotics or not, use of a Fastidious Antimicrobial Neutralisation (FAN)-aerobic B/C bottle to culture for the pathogens may improve culture yield. This was seen in a Korean study^14^ where the culture-positivity yield was significantly improved when a FAN B/C bottle was used as opposed to inoculation directly onto agar media. Also, further comparisons are required to determine if inoculating sample into a B/C bottle at the patient bedside will improve culture yield as opposed to performing this in the laboratory. Culture-negative yields of < 15% was observed when samples were inoculated into B/C bottles at the bedside.^14^ In another study, it was reported that centrifugation or 4 h-sedimentation did not improve time to positivity of culture results.^15^

## Conclusion

There are still several unknowns on what can improve the culture yield of PD fluid processing. More evaluations are warranted around this test method.
